# Medical and nursing students’ satisfaction with e-learning platforms during the COVID-19 pandemic: Initial findings of an experimental project in China

**DOI:** 10.1016/j.heliyon.2024.e26233

**Published:** 2024-02-15

**Authors:** Suting Chen, Mariana Morgado, Haozhe Jiang, José João Mendes, Jia Guan, Luís Proença

**Affiliations:** a Shanghai Jian Qiao University, Shanghai 201306, China; b Clinical Research Unit (CRU), Egas Moniz Center for Interdisciplinary Research (CiiEM); Egas Moniz School of Health & Science, Campus Universitário, Quinta da Granja, 2829-511 Caparica, Almada, Portugal; c College of Education, Zhejiang University, Hangzhou 310058, China; d Center for Educational Technology and Resource Development, Ministry of Education (National Center for Educational Technology, NCET), Beijing 100031, China

**Keywords:** Computer self-efficacy, COVID-19 pandemic, Medical and nursing education, Technology satisfaction model

## Abstract

Satisfaction with learning management systems (LMSs) is an essential indicator of students' e-learning experiences and reflects the quality of e-learning. Applying the technology satisfaction model, the present study aimed to investigate medical and nursing students' satisfaction with LMSs and its predictors. We conducted our survey at a medical university located in East China and received a total of 329 effective responses. Structural equation modelling was used to analyse the data. Our findings confirmed that perceived ease of use and perceived usefulness were two direct predictors of medical and nursing students' satisfaction with LMSs. Furthermore, the influence of perceived usefulness on satisfaction was more powerful than that of perceived ease of use. This study also substantiated that computer self-efficacy and perceived ease of use can indirectly impact medical and nursing students' satisfaction with LMSs. Our research effectively links the theoretical hypotheses with empirical findings, highlighting the central role of Computer Self-Efficacy (CSE), perceived ease of use, and perceived usefulness in shaping medical and nursing students' satisfaction with LMSs. Our findings contributed to the understanding of the technology satisfaction model and medical and nursing students’ e-learning during the COVID-19 pandemic.

## 1 Introduction

### 1.1 Background

The outbreak of the COVID-19 pandemic has led to unprecedented changes in the way students around the world learn [[1], [2], [3], [4], [5]]. In this context, the sudden transition from traditional face-to-face learning to e-learning has created many challenges for university students, particularly in the field of medical and nursing education [3,6,7]. To facilitate the improvement of e-learning experiences and address these challenges, it is imperative to gain a thorough understanding of how medical and nursing students perceive and engage with e-learning during this critical period [8]. Learning Management Systems (LMSs) serve as central platforms to support e-learning initiatives (for more details, see Refs. [[1], [2], [3]]). Student satisfaction with LMSs plays a fundamental role in measuring the overall quality of e-learning experiences [1,2,9]. Assessing student satisfaction with LMSs and identifying the factors that influence it can provide critical insights into the effectiveness of e-learning and identify areas within LMSs that require attention and improvement. Importantly, during the COVID-19 pandemic, where e-learning has become a primary mode of education, an increased focus on student satisfaction with LMSs is warranted [1,2,8,10].

In parallel with global education changes, China has been actively promoting STEM (Science, Technology, Engineering and Mathematics) education reforms, with a particular focus on medical and nursing education [[11], [12], [13]]. Various initiatives have been launched in China to improve medical and nursing education systems, including the integration of telemedicine and videoconferencing systems, the development of massive open online courses (MOOCs), and the introduction of virtual reality simulations [14,15]. However, despite these efforts, some Chinese medical and nursing students encountered challenges and reported suboptimal e-learning experiences, particularly during the COVID-19 pandemic [16].

Medical and nursing education differs significantly from other disciplines due to the practical nature of clinical skills development and the need for structured curricula in a clinical environment [14,17]. It is also argued that “modern medical education encompasses a well-thought-out training system that covers highly structured curricula in a variety of preclinical and clinical environments” [14] (p. 2). These unique requirements present a significant challenge to the full implementation of e-learning through LMSs [14]. As a result, medical and nursing students often have higher demands and expectations of LMSs, when compared with students of other majors. Given the reported problems with medical and nursing students' e-learning experiences during the pandemic [16].

The emergence of e-learning in medical and nursing education has brought about transformative changes in the way students acquire knowledge and skills. Understanding student satisfaction with e-learning in this specific context is of paramount importance. While the theoretical base in this area is still developing, existing models such as the Technology Acceptance Model (TAM) [18] and its extensions, such as the Unified Theory of Acceptance and Use of Technology (UTAUT) [19], and the Technology Satisfaction Model (TSM) [20] provide valuable frameworks for understanding satisfaction with e-learning. The TAM, rooted in technology acceptance and user satisfaction, considers factors such as perceived ease of use and perceived usefulness. The TSM, on the other hand, provides a structured approach to understanding technology satisfaction.

### 1.2 Previous studies and limitations

Previous studies investigating the e-learning experiences of medical and nursing students have provided valuable insights. However, they have had certain limitations that our research seeks to address. For example, some of these studies focused primarily on assessing e-learning satisfaction in a general educational context, inadvertently overlooking the specific needs of medical and nursing students [18]. Conversely, other studies examined e-learning experiences in the midst of the COVID-19 pandemic, but did not use a comprehensive theoretical framework to analyse the factors influencing satisfaction [19]. To address these research gaps and challenges, this study uses the Technology Satisfaction Model (TSM), a powerful framework that has been widely used to understand individuals' technology satisfaction in Asian contexts. The technology satisfaction model (TSM) is one powerful and effective model which explains individuals’ technology satisfaction in Asian contexts [1,[20], [21], [22], [23], [24]]. Furthermore, while some studies have explored the challenges of e-learning, they have not extensively explored the determinants of satisfaction [25]. In contrast, our research seeks to fill this gap through a thorough examination of key determinants, including computer self-efficacy (CSE), perceived ease of use (PEOU), and perceived usefulness (PU). These determinants will be rigorously investigated in the unique context of medical and nursing students' e-learning experiences during the COVID-19 pandemic.

The limitations identified in previous research have important practical implications for both educators and policy makers in the field of medical and nursing education. By recognising the overlooking of specific needs within the e-learning experiences of medical and nursing students, we highlight the importance of tailoring educational approaches to meet the unique demands of these students. Addressing this limitation is critical to ensuring that medical and nursing students receive effective and engaging online education. Furthermore, the limited exploration of the determinants of satisfaction in previous studies calls for a more comprehensive understanding of the factors that influence student satisfaction. Such knowledge is crucial for educators and institutions seeking to improve the quality of e-learning experiences and student outcomes. Our research aims to address these limitations and contribute to a more tailored understanding of this unique group's e-learning experiences during the COVID-19 pandemic.

### 1.3 Research objectives

The primary objective of this research is to investigate the satisfaction of Chinese medical and nursing students with e-learning during the COVID-19 pandemic. We aim to uncover the direct and indirect determinants of their e-learning satisfaction using the TSM. By shedding light on their experiences and satisfaction levels, this study not only contributes to a deeper understanding of how medical and nursing students adapt to e-learning during a global health crisis but also holds the potential to enhance the quality of healthcare education and, consequently, the safety of future healthcare delivery. Ultimately, the significance of this research lies in its potential to safeguard patient well-being and advance the field of medical and nursing education in the rapidly evolving digital landscape, bridging a critical gap in our understanding of the challenges faced by students in the era of e-learning and the COVID-19 pandemic.

### 1.4 Significance of the study

This research is noteworthy for several important reasons. It uniquely focuses on medical and nursing students, addressing their specific e-learning challenges and needs. Conducted during the disruptive COVID-19 pandemic, it explores how these students adapt to e-learning in a global health crisis. The study uses the Technology Satisfaction Model (TSM) to rigorously understand their satisfaction with e-learning. In particular, the application of the TSM to e-learning for medical and nursing students during a pandemic is innovative. Furthermore, this research has the potential to have a significant impact on healthcare education, improving its quality and influencing the safety and effectiveness of future healthcare provision. In summary, its specific focus, pandemic context, theoretical framework and potential to influence health education make it a pioneering contribution to the field.

## 2 Theoretical model

**Fig. 1:**
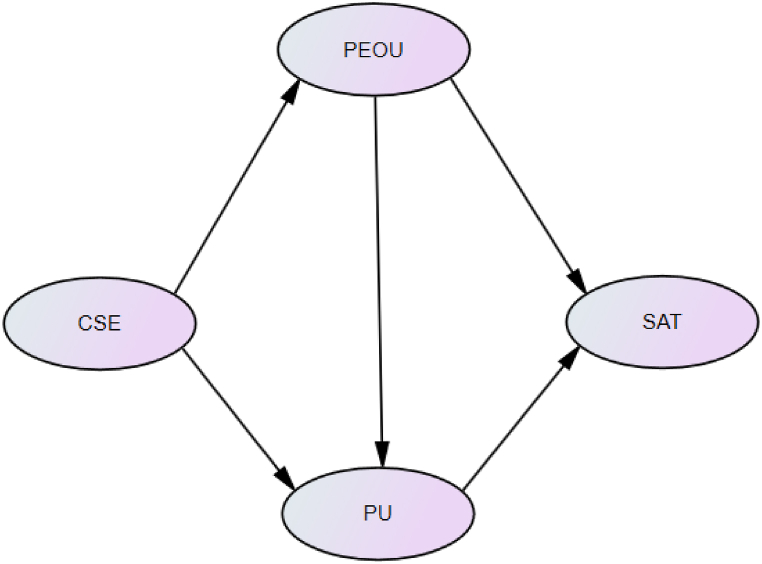
Tsm [1,[20], [21], [22], [23], [24]].

Students' technology satisfaction is the degree to which “the use of technology is consistent with existing values, needs and student experiences” [22] (p. 4). Students' technology satisfaction has been considered as an important indicator as it greatly impacts students' overall experiences of using certain type(s) of technology [25]. In the settings of Asian higher education, TSM is one important theoretical model which can powerfully explain students’ technology satisfaction [1,[20], [21], [22], [23], [24]]. This model was proposed in 2014 [20] and has been cross-validated in several representative Asian countries such as Malaysia [21], Pakistan [23] and China [22]. TSM integrates the social cognitive theory [26] into the technology acceptance model [27] to explain technology satisfaction (SAT) with three factors, namely computer self-efficacy (CSE), perceived ease of use (PEOU) and perceived usefulness (PU) [20]. Specifically, two elements of the technology satisfaction model (i.e., CSE and SAT) derive from the social cognitive theory [26] and the other two elements (i.e., PEOU and perceived usefulness) derive from the technology acceptance model [27]. In doing so, the technology satisfaction model makes up for one defect of the traditional technology acceptance model [27], which is the ignorance of psychological factors like CSE [28,29] and SAT [28,30]. Hence, we decided to base our study on TSM. TSM suggests that PEOU and PU are two immediate antecedents of SAT while CSE is one indirect antecedents of SAT [1,[20], [21], [22], [23], [24]]. Five hypotheses constitute TSM (see Fig. 1).

While TSM has been applied in various settings, its relevance to medical and nursing students' e-learning experiences during the pandemic has yet to be explored. One significant gap in the literature pertains to the limited understanding of medical and nursing students' e-learning satisfaction during the pandemic. Objective 1 directly addresses this gap by focusing on this specific group of students. Previous research has primarily explored e-learning satisfaction in broader educational contexts, overlooking the unique needs and challenges faced by medical and nursing students. Achieving this objective will provide crucial insights into the satisfaction levels of these students, shedding light on their experiences during the pandemic. One significant gap in the literature pertains to the limited understanding of medical and nursing students' e-learning satisfaction during the pandemic. Objective 1 directly addresses this gap by focusing on this specific group of students. Previous research has primarily explored e-learning satisfaction in broader educational contexts, overlooking the unique needs and challenges faced by medical and nursing students. Achieving this objective will provide crucial insights into the satisfaction levels of these students, shedding light on their experiences during the pandemic. Another gap in the literature is the lack of clarity regarding the determinants of e-learning satisfaction for medical and nursing students. Objective 2 seeks to bridge this gap by examining key factors, such as CSE, PEOU, and PU, within the context of medical and nursing students' e-learning experiences during the pandemic. By doing so, this research aims to uncover the unique determinants of satisfaction for this specific group of students, enriching our understanding of the factors influencing their e-learning experiences. The third gap in the literature centres on the need for comprehensive insights into medical and nursing students' e-learning experiences. Objective 3 directly addresses this gap by conducting a thorough examination of their e-learning satisfaction, determinants of satisfaction, and overall experiences during the pandemic. This objective aims to provide a nuanced understanding of the challenges and needs specific to medical and nursing students in the context of e-learning, filling a critical void in the existing literature.

### 2.1 Hypotheses of TSM

Self-efficacy is used to describe “people's beliefs about their capabilities to produce designated levels of performance that exercise influence over events that affect their lives” in social cognitive theory [26] (p. 71). Drawing from this definition, in the education field, CSE can be used to describe a “student's beliefs in their capabilities to use a computer for their learning and research” [21] (p. 57). The variable CSE has seldom been operationally defined and investigated when it comes to medical and nursing students' e-learning. In this study, we conceptualized it as medical and nursing students' beliefs in their capabilities to use LMSs for their medical and nursing learning and research.

Prior studies have shown that CSE can directly influence individuals' PEOU and PU of certain type(s) of technology [[20], [21], [22], [23], [24],[31], [32], [33], [34], [35]]. More importantly, these influences have also been verified in the context of using LMSs [1,2]. In other words, university students may find it easier and more helpful to use LMSs if they have higher levels of CSE [1,2]. However, considering the particularity of medical and nursing e-learning [14,16,17], whether these two influences exist when it comes to medical and nursing students’ e-learning is not well-documented. We hypothesize.

Hypothesis 1 Medical and nursing students' CSE will directly influence their PEOU of LMSs.

Hypothesis 2 Medical and nursing students' CSE will directly influence their PU of LMSs.

PEOU and PU are two variables which originate from the technology acceptance model [27]. They can be adapted to the e-learning contexts, and respectively referred to as “students' perception of the benefits of using” LMSs [1] (p. 6752) and “students' perception of how easy or difficult it is to use” LMSs [1] (p. 6753). As for the relationships of PEOU and PU, previous studies have not reached consistent conclusions. Most studies have claimed that PEOU significantly impacts PU [[36], [37], [38], [39], [40], [41]]. For instance, it was found that Togolese citizens' PEOU of e-government significantly impacted their PU [36]. For another instance, it was confirmed that Jordanian university students' PEOU of Massive Open Online Courses (MOOCs) significantly impacted their PU. However, some studies do not support such a significant effect [40]. For instance, a recent study in India showed that educators' PEOU of digital technologies did not significantly impact their PU [42]. To date, the relationship between medical and nursing students’ PEOU and PU of LMSs has not been well discussed. We hypothesize.

Hypothesis 3 Medical and nursing students' PEOU of LMSs will directly influence their PU of LMSs.

Prior studies have also pointed out that PEOU and PU are two immediate predictors of technology SAT [[20], [21], [22], [23], [24]]. This has also been certified in the e-learning contexts [1,2]. In other words, if students find it easy and helpful to use LMSs, they will feel satisfied. However, considering the particularity of medical and nursing e-learning [14,16,17], whether these two effects exist among medical and nursing students needs more examination. We hypothesize.

Hypothesis 4 Medical and nursing students' PEOU of LMSs will directly influence their SAT with LMSs.

Hypothesis 5 Medical and nursing students' PU of LMSs will directly influence their SAT with LMSs.

## 3 Materials and methods

### 3.1 Procedure and sample

We conducted this study at a medical university located in East China. During the COVID-19 pandemic, all the students in the medical university experienced e-learning for at least one semester. The LMSs used in the medical university not only have general features (including the capability to disseminate knowledge, assess learners' competence, record learners’ attainment, and facilitate the communications among social communities; for more details of the general features, see Refs. [1,2,43,44]), but also enables students to watch various videos of medical experiments. The most frequently used e-learning platform is called Chaoxing®.

This study obtained approval from the Research Ethics Committee before data collection. Following the principles of data protection, no information that may lead to the identification of our participants was collected. We aimed to collect at least 300 effective responses so that the sample size could be adequate for structural equation modelling analysis [45]. We randomly selected several teaching administrators and invited them to distribute the online recruitment information among their students. Students who were interested in our research could voluntarily click the questionnaire link and fill in the scales online. After removing incomplete ones, we received a total of 329 effective responses. The response rate was 88.92%. All the participants were medical or nursing students. Among them, 38% were male students and 62% were female students. 8.5% were 17 or 18 years old, 43.2% were 19 or 20 years old, 46.2% were 21 or 22 years old, and 2.1% were 23 or 24 years old.

### 3.2 Data collection tool and data analysis

Our data collection tool was originated from Islam's study [32,46], and translated into Chinese by Jiang et al. [1,2], which contained four constructs, namely PEOU, PU, CSE and SAT. Six-point Likert scales were used. According to the literature, six-point Likert scales can help respondents “escape the in between scenario” (e.g., neutral/undecided) so that we can be informed of respondents' exact perceptions of LMSs [47] (p. 184). After the formal data collection, preliminary data analysis was conducted. Some items were removed due do their low factor loadings were low or multicollinearity was detected. A total of twenty items were included in the final data analysis. These items are shown in the Appendix.

As for the data analysis, we applied a structural equation modelling approach [48] using the software named IBM SPSS AMOS 22. Structural equation modelling is widely used to test the direct and indirect relationships among a series of variables [48]. Notably, the structural equation model is made up of the measurement model and the structural model [48]. To examine the validity and reliability of the research instrument (see the Appendix), we tested the measurement model (the validity and reliability of the research instrument is shown in the Results section). To examine the effects of CSE, PEOU and PU on SAT, we tested the structural model.

To evaluate the model fit, we chose to use some goodness-of-fit indices, including the chi-square to degree of freedom ratio (χ2/df), the comparative fit index (CFI), the Tucker-Lewis index, and the root mean square error of approximation (RMSEA) [48]. Specifically, it is recommended χ2/df ≤ 5, CFI ≥0.90, TLI ≥0.90, RMSEA ≤0.08.

## 4 Results

### 4.1 The results of the measurement model

To examine the validity and reliability of the research instrument, we tested the measurement model. The measurement model consisted of four constructs with twenty items (for the details, see the Appendix). The AMOS output showed that it fitted the data well [45,49], with χ^2^ = 476.748, *df* = 164, *p* = 0.000, CFI = 0.954, TLI = 0.947, and RMSEA = 0.076.^2^ Table 1 summarizes the results of the measurement model.

**Table 1:** Summary of the results of the measurement model.

Construct	Item	M	SD	FL	α	CR	AVE
CSE	CSE1	5.116	0.9167	0.807	0.921	0.924	0.752
	CSE5	4.736	1.1343	0.837			
	CSE6	4.906	1.0001	0.912			
	CSE7	4.854	0.9800	0.908			
PEOU	PEOU1	4.802	1.0737	0.815	0.943	0.944	0.737
	PEOU5	4.468	1.1288	0.861			
	PEOU6	4.617	1.1366	0.781			
	PEOU7	4.495	1.1453	0.902			
	PEOU8	4.751	1.0557	0.914			
	PEOU10	4.757	1.0513	0.870			
PU	PU3	4.581	1.1584	0.860	0.922	0.927	0.680
	PU4	4.091	1.3243	0.633			
	PU5	4.505	1.1400	0.814			
	PU6	4.459	1.1709	0.875			
	PU7	4.334	1.1986	0.871			
	PU10	4.726	1.0554	0.867			
SAT	SAT1	4.881	1.0097	0.931	0.925	0.926	0.759
	SAT2	4.681	1.0023	0.788			
	SAT3	4.845	0.9739	0.856			
	SAT4	4.723	1.0416	0.903			

As for the reliability, it is recommended that the values of the Cronbach's alphas (α) for all constructs should be above 0.70 [50,51]. As shown in Table 1, none of the values of the Cronbach's alphas was lower than 0.9. Therefore, the reliability of our instrument was very satisfactory.

As for the convergent validity, it is recommended that the values of composite reliability (CR) and the average variance extracted (AVE) should be above 0.7 and 0.5, respectively [[49], [50], [51]]. As shown in Table 1, none of the values of CR and AVE was lower than 0.9 and 0.6. Therefore, the convergent validity of our instrument was very satisfactory.

As for the discriminant validity, we followed [52]'s rules to ensure that the square roots of the AVEs were larger than the correlations.

Notably, our findings showed that 1 participant (0.31%) was strongly dissatisfied with e-learning platforms, 4 participants (1.23%) were moderately dissatisfied with e-learning platforms, 14 participants (4.26%) were slightly dissatisfied with e-learning platforms, 60 participants (18.24%) were slightly satisfied with e-learning platforms, 149 participants (45.29%) were moderately satisfied with e-learning platforms, and 101 participants (30.70%) were moderately satisfied with e-learning platforms.

### 4.2 The results of the structural model

**Fig. 2:**
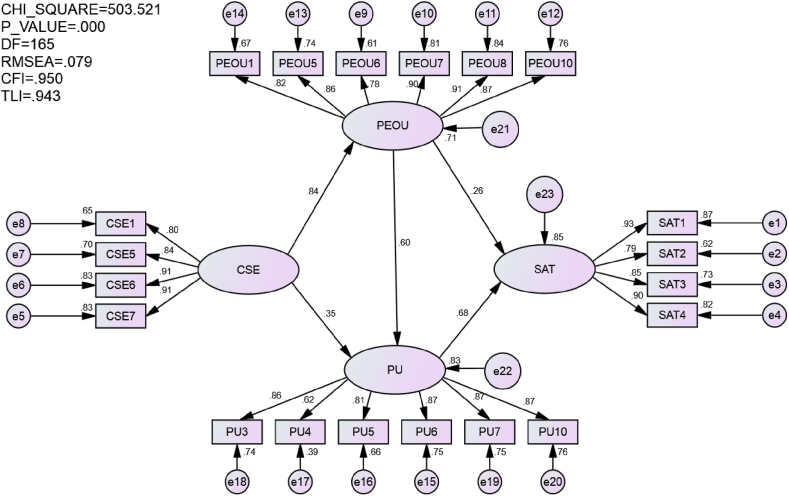
The results of the structural model.

To examine the effects of CSE, PEOU and PU on SAT, we tested the structural model. As shown in Fig. 2, the AMOS output showed that the structural model fitted the data well [43,47], with χ^2^ = 503.521, *df* = 165, *p* = 0.000, CFI = 0.950, TLI = 0.943, and RMSEA = 0.079. In the following two subsections, the direct effects and indirect effects within the model were respectively described.

#### 4.2.1 The results of the direct effects

The results of the direct effects are summarized in Table 2. It was confirmed that medical and nursing students' CSE significantly impacted their PEOU (*β =* 0.842, *p* < 0.001) and PU (*β =* 0.349, *p* < 0.001) of LMSs. Medical and nursing students' PEOU significantly impacted their PU (*β =* 0.597, *p* < 0.001) of LMSs. Medical and nursing students’ SAT with LMSs was significantly affected by their PEOU (*β =* 0.260, *p* < 0.005) and PU (*β =* 0.685, *p* < 0.001). Therefore, all the five hypotheses could be accepted.

**Table 2:** The results of the direct effects.

Hypothesis	Path	*β*	C.R.	Result
Hypothesis 1	CSE→PEOU	0.842***	15.345	Accepted
Hypothesis 2	CSE→PU	0.349***	5.489	Accepted
Hypothesis 3	PEOU→PU	0.597***	8.533	Accepted
Hypothesis 4	PEOU→SAT	0.260**	3.116	Accepted
Hypothesis 5	PU→SAT	0.685***	7.843	Accepted

*Note: p* < 0.001 (***), *p* < 0.005 (**), *p* < 0.01 (*).

It was worth noting that our findings certified that PEOU and PU were two direct antecedents of medical and nursing students’ SAT with LMSs. Particularly, the effect of perceived of usefulness on SAT was stronger than that of PEOU.

#### 4.2.2 The results of the indirect effects

The total indirect effects of medical and nursing students’ CSE on their SAT with LMSs were 0.802 (*p* < 0.005). Specifically, CSE indirectly impacted SAT via three paths: CSE→PEOU→SAT, CSE→PU→SAT, and CSE→PEOU→PU→SAT. The indirect effects were respectively 0.219, 0.239, and 0.344.

The indirect effect of medical and nursing students’ PEOU on their SAT with LMSs was 0.409 (*p* < 0.005). Specifically, PEOU indirectly impacted SAT via one path: PEOU→PU→SAT. Considering that the direct effect of PEOU on SAT was 0.260 (*p* < 0.005), the total effects of PEOU on SAT were 0.669 (*p* < 0.005).

## 5 Discussion

As an essential indicator of e-learning effectiveness and quality, e-learning SAT has not received enough attention [1,2,8,10]. Our primary research objective was to investigate the satisfaction levels of Chinese medical and nursing students with e-learning during the COVID-19 pandemic. By accomplishing this objective, we aimed to identify both the direct and indirect determinants of their e-learning satisfaction employing the Technology Satisfaction Model (TSM). Our study successfully achieved these aims, unravelling critical insights into how medical and nursing students adapt to e-learning amid a global health crisis.

The SAT is a key indicator of the quality and effectiveness of e-learning. However, it has often been underemphasised in the literature. Our study contributes significantly to the understanding of e-learning SAT by focusing on the experiences of medical and nursing students during the COVID-19 pandemic. We found that perceived ease of use (PEOU) and perceived usefulness (PU) have a direct influence on SAT of medical and nursing students. In addition, our findings revealed that CSE and PEOU indirectly influence SAT. An outstanding finding was the confirmation that CSE emerged as the most influential predictor of AT, a revelation that has rarely been documented.

A noteworthy contribution of our study was the application of the Technology Satisfaction Model (TSM) to a previously unexplored context—the e-learning experiences of medical and nursing students. Given the unique demands and requirements of medical and nursing education, this extension of TSM's applicability underscores its robust explanatory power and adaptability across diverse educational settings. (for more details, see the Introduction section and [14,16,17]).

We shed light on the hierarchy of influential factors within the TSM framework. Our findings are consistent with previous studies that identified PEOU and PU as direct predictors of technology SAT. However, our research also revealed that CSE and PEOU act as indirect predictors of technology SAT. Importantly, we quantified the total effect of each factor on e-learning SAT among medical and nursing students. Specifically, PU had a total direct effect of 0.685, CSE had a total indirect effect of 0.802, and PEOU had a total effect of 0.669 (consisting of direct effects of 0.260 and indirect effects of 0.409). We have therefore conclusively established that CSE is the most powerful predictor of these factors [[20], [21], [22], [23], [24]].

Finally, our findings were instructive for medical and nursing e-learning during the COVID-19 pandemic. It is reported that “the shift to online systems poses a few challenges for medical education” [14] (p. 3). Prior studies also found that many medical and nursing students were unfamiliarity with the LMSs and lack of technical support [53,54]. Therefore, it is urgent to have a deep understanding of medical and nursing students' e-learning experiences and let medical and nursing students be free of technical troubles of LMSs. Our findings show that CSE was the most influential factor of SAT among the three factors. Hence, it is very necessary to develop students' computer-related capabilities. Universities can also hold some workshops to help medical and nursing students develop their systems of using LMSs during the COVID-19 pandemic. Furthermore, to enhance medical and nursing students' PU, LMSs can develop more helpful functions. For instance, as some investigations suggested, medical and nursing students hoped LMSs could introduce “real-time chat tools” to “extend students' questions beyond the classroom” so that students were able to “ask questions at any time when they find problems in their studies, and get detailed answers in a short time”, and also “view the questions asked by others” [14] (p. 4). Besides, to enhance medical and nursing students’ PEOU, LMSs can also simplify their interface and make user friendly as one of their features.

Inevitably, this study has some limitations. For instance, we adopted the technology satisfaction model as our theoretical framework and discussed e-learning SAT from a technical angle. However, other elements such as curricular design and teachers were not involved. Further studies can focus on medical and nursing students' e-learning from different angles, especially their SAT with the highly-structured medical and nursing education courses [14]. Future studies could explore these additional factors to provide a more comprehensive model to predict e-learning satisfaction. Future studies could explore these additional factors to provide a more comprehensive understanding of e-learning satisfaction. For another instance, although our sample size was large enough, only one medical and nursing university was selected. Future studies should include more medical and nursing universities to examine our research findings. By the way, following the rules of Research Ethics Committee, this study employed voluntary response sampling.^3^ This may allow bias to creep in on the results and skew data, impacting the overall representativeness of our findings. In other words, as we used voluntary response, only those who were inclined to respond were recruited in our study. This means some groups of students (e.g., students who held negative opinions on e-learning) may not be represented in this study. In addition, it is pointed out that teachers may not be able to provide enough supervisory via LMSs [55]. It is necessary to scrutinize its influence on medical and nursing students’ acquirement of clinic capabilities and related SAT.

Considering the replicability limitations of this study, it's important to acknowledge factors impacting its reproducibility. The study's focus on Chinese medical and nursing students during the COVID-19 pandemic is deeply tied to China's unique context, including cultural, educational, and institutional factors. Replicators in different settings must be mindful of these nuances. The timing during the pandemic, a period of educational disruption, is significant. Replicating the study outside this context may yield different results due to the evolving pandemic and data collection timing. The rapid evolution of e-learning technology poses a challenge. Learning systems and tools may have changed. Replicators should adapt to the current technological landscape. The sample was Chinese medical and nursing students, potentially introducing selection bias. Efforts must ensure a diverse and representative sample. Generalizability to other healthcare-related degrees or educational settings may be limited. Replicators should assess applicability, considering variations in curricular design and policies. External factors like government policies may have influenced outcomes. Replicators should consider such factors in their context. Lastly, methodology may require specific resources. Replicators should evaluate feasibility given available resources. In conclusion, replicability should consider contextual, temporal, technological, and methodological factors. Researchers replicating or building upon this study should be mindful of these limitations.

In conclusion, our study makes significant progress in understanding SAT, particularly in the context of medical and nursing education during the COVID-19 pandemic. Our findings highlight the pivotal role of CSE and provide practical insights for educational institutions. While acknowledging the limitations of the study, it serves as a foundation for future research in this rapidly evolving digital educational landscape, filling a critical gap in our understanding of the challenges faced by students in the era of e-learning and the COVID-19 pandemic. Researchers are encouraged to build on this work, considering the evolving technological and educational landscape.

## 6 Conclusions

In conclusion, it was found that 5.78% participants were dissatisfied with e-learning platforms overall, and 94.22% participants were satisfied with e-learning platforms. Our study using the Technology Satisfaction Model (TSM) has provided valuable insights into the factors influencing medical and nursing students' satisfaction with Learning Management Systems (LMSs) during the COVID-19 pandemic. We confirmed that Perceived Ease of Use (PEOU) and Perceived Usefulness (PU) have a direct impact on student satisfaction with LMSs, with PU having a stronger influence. In addition, we found that computer self-efficacy (CSE) and PEOU indirectly influence satisfaction.

Our contributions are threefold. First, we have extended the application of TSM to the unique context of medical and nursing e-learning (for more details, see the Introduction section and [14,16,17]), demonstrating its effectiveness in this domain. This extension highlights the versatility of TSM as a robust theoretical framework. Secondly, our study uniquely identified CSE as the most influential factor in determining satisfaction, despite its indirect effects. This finding represents a novel contribution to the existing literature. Finally, our research provides valuable guidance for medical and nursing e-learning in the challenging times of the COVID-19 pandemic.

By shedding light on the factors that influence medical and nursing students' satisfaction with e-learning, our study aims to improve the quality of healthcare education and thus contribute to the safety of future healthcare. In an ever-evolving digital landscape, filling the gap in our understanding of student challenges in the e-learning era is paramount. Our study highlights the importance of addressing these challenges and advancing medical and nursing education in a rapidly changing environment.

In conclusion, our research represents a critical step towards improving the landscape of medical and nursing education in the digital age, particularly during unprecedented challenges such as the COVID-19 pandemic. By uncovering the central role of perceived ease of use, perceived usefulness, and computer self-efficacy in shaping e-learning satisfaction, we provide a basis for refining educational strategies and platforms.

The significance of our work extends beyond academia, with the potential to safeguard patient welfare by ensuring that healthcare professionals receive high quality education. As we continue to navigate the ever-evolving digital landscape, addressing the challenges students face in e-learning remains paramount. This study serves as a catalyst for innovation, pushing the boundaries of medical and nursing education to new heights.

Essentially, our findings point the way forward and encourage the development of more effective and learner-centred e-learning environments. As the world of healthcare continues to evolve, so too must our educational methods. Our research contributes to this imperative evolution, ultimately aiming for a future where healthcare education meets the demands of our dynamic world and ensures the highest standards of patient care and safety.

## Data availability statement

Data will be made available upon reasonable request.

## Ethics statement

This study was reviewed and approved by the Research Management Centre for Non-Clinical Faculties at Shanghai Jian Qiao University, with the approval number: HR 260–2020.

## Additional information

No additional information is available for this paper.

## Funding

This work was supported by the National Natural Science Foundation of China (Grant number: 72374072).

## Institutional review board statement

This study had obtained the ethical endorsement from the universities and Research Management Centre for Non-Clinical Faculties at Shanghai Jian Qiao University (No.: HR 260–2020).

## Informed consent statement

Informed consent was obtained from all subjects involved in the study.

## CRediT authorship contribution statement

**Suting Chen:** Writing – original draft, Visualization, Validation, Software, Resources, Investigation, Funding acquisition, Data curation. **Mariana Morgado:** Writing – review & editing, Writing – original draft. **Haozhe Jiang:** Writing – review & editing, Writing – original draft, Visualization, Validation, Supervision, Software, Resources, Methodology, Investigation, Formal analysis, Data curation, Conceptualization. **José João Mendes:** Writing – review & editing. **Jia Guan:** Visualization, Validation, Project administration, Investigation. **Luís Proença:** Writing – review & editing.

## Declaration of competing interest

The authors declare that they have no known competing financial interests or personal relationships that could have appeared to influence the work reported in this paper.

## Appendix

Questionnaire used in the formal data analysis. .

Back Matter.

## Footnotes

2 the comparative fit index (CFI), the Tucker–Lewis Index (TLI), and the root mean square error of approximation (RMSEA).

3 It is impossible and unethical to ask students who were not interested in our study to complete our survey. Therefore, we employed voluntary response sampling.
